# Supplementary material for: Flight power muscles have a coordinated, causal role in controlling hawkmoth pitch turns
Source: J Exp Biol. 2024 Dec 18;227(24):jeb246840. doi: 10.1242/jeb.246840 (PMC11698061; doi:10.1242/jeb.246840)
Supplement: Supplementary information [file jexbio-227-246840-s1.pdf]

Supplementary Materials and Methods

1 Nonlinear Kernel CCA

The CCA analysis of the main text is inherently linear in nature, constituting a reprojection of the pitch torque waveforms guided to maximize cross-covariance with induced DLM phase. It is possible that some of the key features of the pitch torque which varied with DLM phase might be inherently nonlinear. So, it is relevant to assess to what degree a nonlinear dual-dimensionality reduction method could perform better, and whether a nonlinear method extracts fundamentally different features from the pitch torque data. To that end, we performed kernel CCA (KCCA), a variant of the traditional CCA with an nonlinear kernel transform of the component variables. See (Akaho, 2006) and (Hardoon et al., 2004) for review, examples, and deeper coverage of kernel CCA. In brief, in KCCA both sets of data $\boldsymbol{\mathcal{T}}$ and $\boldsymbol{Y}$ are projected into a high-dimensional reproducing kernel Hilbert space using a kernel function $\phi(X)$ and traditional CCA is performed on the kernel space representations of the data $\phi(\boldsymbol{\mathcal{T}})$ and $\phi(\boldsymbol{Y})$. By performing the normally linear CCA in this high-dimensional space, it can capture transformations which would be nonlinear in the original basis, with minimal additional computational complexity compared to normal CCA.

KCCA does require a choice of a positive definite kernel function $\phi(X)$, and results can be sensitive to both the choice of this function and any constant parameters defining the function. We opted to use the radial basis function, also known a the Gaussian kernel, as our kernel, defined by

$$\phi(x, y) = e^{-\gamma||x-y||^2} \tag{1}$$

as it is commonly used, implicitly defines an infinite-dimensional Hilbert space, and requires choice of only a single parameter γ which defines the width of the radial Gaussian.

One downside of KCCA is that kernel functions are rarely invertible, so there does not always exist a well defined function $\phi^{-1}(X)$. This has the consequence of making it relatively easy to find a latent space in which two sets of variables $\boldsymbol{\mathcal{T}}$ and $\boldsymbol{Y}$ are maximally correlated, but moving back from this latent space to reconstruct features in the original data space which the KCCA extracted, as we do in the main text using Eq. (1), is not directly possible.

To evaluate the features extracted by KCCA, and how much of the variance in pitch torque $\boldsymbol{\mathcal{T}}$ they explain, we followed a common solution first proposed by Bakır et al (Bakır

Journal of Experimental Biology • Supplementary information

et al., 2004) and adapted from the implementation in scikit-learn's KernelPCA class (Pedregosa et al., 2011). In this technique, rather than try to evaluate $\phi^{-1}(X)$, a function Γ is estimated which approximately satisfies $\Gamma(\phi(\boldsymbol{\mathcal{T}}_i)) = \boldsymbol{\mathcal{T}}_i$ for all n wingstrokes $i = 1, \ldots, n$, in our case using a kernel ridge regression.

The results of running KCCA and estimating an inverse transform to reconstruct pitch torque features is shown in Fig. S1. To ensure the KCCA explained as much variance in pitch torque as possible, the parameter $\gamma = 0.4018$ was chosen as it maximizes the mean r^2 across moths between pitch torque reconstructions and the actual pitch torque waveforms, as shown in Fig. S2. Compared to the linear CCA, this nonlinear method explained on average 7.4% more variance in pitch torque. Crucially, however, it did not extract any features of the pitch torque waveforms that differed dramatically from the features found via linear CCA. While the nonlinear method was able to extract pitch torque waveforms that did not simply rescale based on induced DLM timing, both methods extracted features consisting of 3-4 major peaks with a pronounced phase shift in the largest peak associated with later induced DLM phase. This indicates that there is an underlying relationship between induced DLM phase and pitch torque which is robust to the particular method of feature identification. while a nonlinear method may be able to explain slightly more variance, it is unlikely to extract significantly different features of pitch torque, is more complicated, and does not allow for explicit invertibility.

Journal of Experimental Biology • Supplementary information

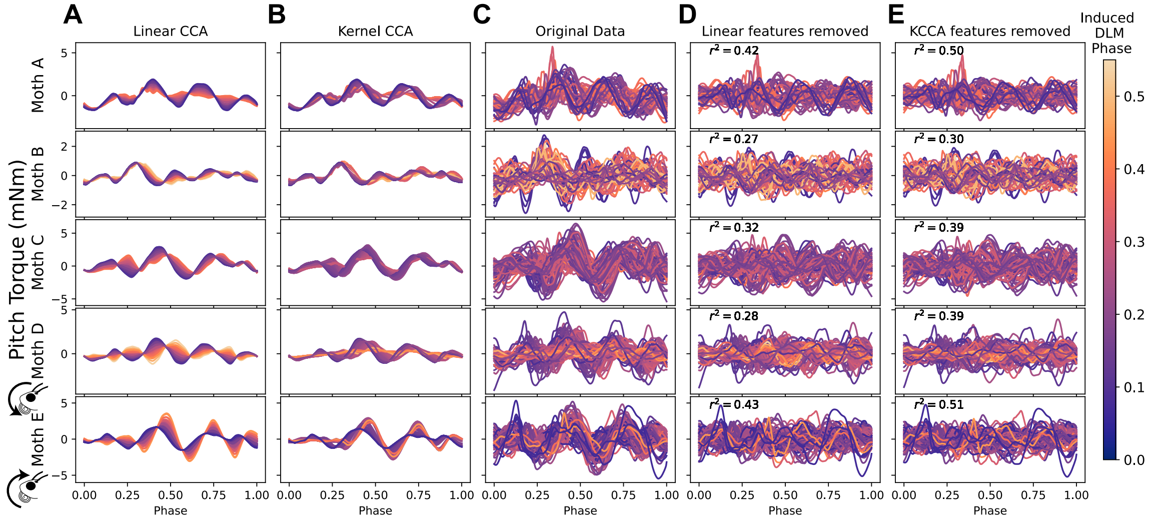

Fig. S1. Pitch torque features extracted from kernel and regular CCA. (**A**) Linear CCA feature reconstructions of pitch torque in stimulation wingstroke, colored by evoked DLM phase. (**B**) Nonlinear kernel CCA feature reconstructions, colored on the same scale as (A-E) (**C**) Actual data of pitch torque in stimulation wingstroke (**D**) Raw data with linear CCA feature reconstructions subtracted, leaving only pitch torque variance unexplained by linear CCA features. r^2 for feature reconstructions printed for each moth. (**E**) Raw data with nonlinear kernel CCA feature reconstructions subtracted. r^2 for feature reconstructions printed for each moth.

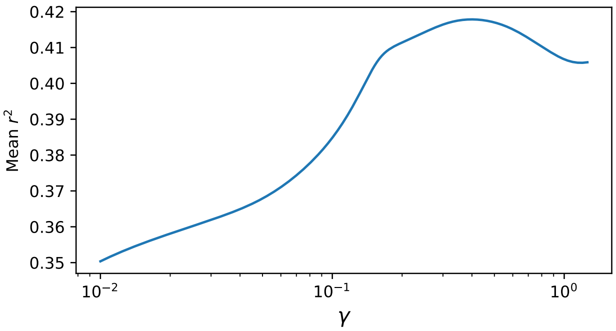

Fig. S2. Parameter search for optimal γ. KCCA is performed between induced DLM phase and pitch torque waveforms with the radial basis function kernel of Eq. (1) across varying values of γ, for all data in dataset. At each γ pitch torque reconstructions are performed and the r^2 of these reconstructions compared to the actual pitch torque data are calculated, for each moth. This line shows the mean r^2 value across all moths, with the peak value being used to select for γ in further analysis.

Journal of Experimental Biology • Supplementary information

Table S1. Results of linear mixed-effects models. Coefficient estimate *beta* and p-value of that estimate for linear mixed-effects models run between each force or torque and induced DLM timing. Rows are bolded if the relationship is statistically significant ($p < 0.05$). Each model was of the form $F_x \sim \beta Y$ where Y is induced DLM phase, and an additional random constant effect is added to account for individual variation. All models were fit using the nlme package (Pinheiro et al., 2020) in the R language (R Core Team, 2020).

Variable	coefficient β	p-value
F_x	-0.000612	0.51
$\boldsymbol{F_y}$	**-0.00627**	**1e-4**
$\boldsymbol{F_z}$	**0.0049**	**0.045**
T_x	-0.012	0.11
T_y	-0.0119	0.89
T_z	0.02	0.62

2 Induced DLM timing and wingstroke-averaged forces and torques

In the main text, DLM timing was shown to induce a within-wingstroke change in pitch torque, yet through the results of Fig. 6 this change was implicated as relatively minor in the presence of the much greater variation in pitch torque present from other sources in the motor program. To further bolster this analysis, we investigated the degree to which wingstroke-averaged forces and torques correlate with induced DLM phase.

As shown in Fig. S3, wingstroke-averaged forces and torques have little clear relationship with induced DLM phase. To measure whether there was any significant linear relationship between induced DLM phase and any of the wingstroke-averaged forces and torques, we ran linear mixed-effects models between induced DLM phase and wingstroke-averaged values for each force and torque with moth as a random constant effect, the results of which are summarized in Table S1. Induced DLM phase was a statistically significant ($p < 0.05$) predictor of only mean F_y (forward force) and F_z (vertical force). For wingstroke-averaged pitch torque, the primary focus of this paper, there was no detectable relationship between stimulation-induced DLM phase and the resulting mean pitch torque. That said, just because there is no significant correlation with mean wingstroke torque, does not mean that DLM phase has no consistent effect. There were clear within-wingstroke changes to pitch torque due to controlled changes to DLM timing which were on their own mechanically relevant (as can be seen in Fig. 4 and Fig. 5). These changes, however, wash out behind the natural variation present in the rest of the motor program over the course of an entire wingstroke.

Journal of Experimental Biology • Supplementary information

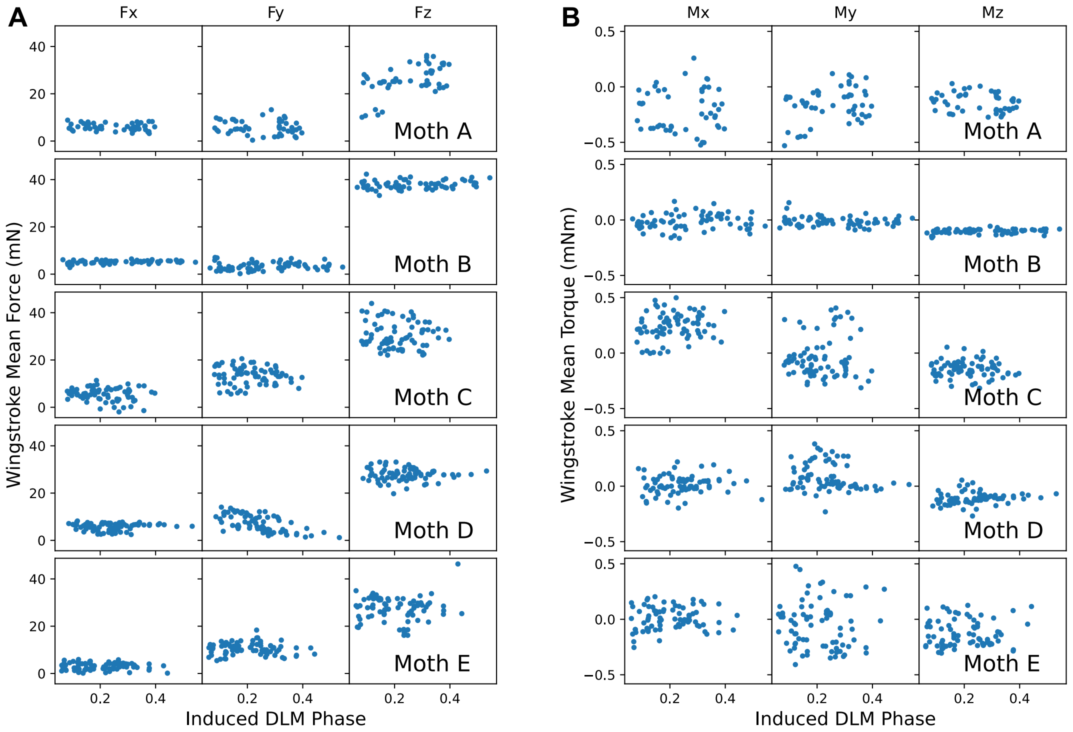

Fig. S3: **Wingstroke mean forces and torques against induced DLM phase**. (**A**) Wingstroke mean forces in X, Y, and Z directions for stimulation wingstrokes plotted against stimulation-induced DLM phase. Directions follow convention of Fig. 1 in the main text (F_y is forward, F_x is lateral, F_z is dorso-ventral). (**B**) Wingstroke mean torques in X, Y, and Z directions for stimulation wingstrokes plotted against stimulation-induced DLM phase. Directions follow convention of Fig. 1 in the main text (T_x is pitch, T_y is roll, T_z is yaw).

Journal of Experimental Biology • Supplementary information

References

Shotaro Akaho. A kernel method for canonical correlation analysis. *arXiv preprint cs/0609071*, 2006.

Gökhan H Bakır, Jason Weston, and Bernhard Schölkopf. Learning to find pre-images. *Advances in neural information processing systems*, 16:449–456, 2004.

David R Hardoon, Sandor Szedmak, and John Shawe-Taylor. Canonical correlation analysis: An overview with application to learning methods. *Neural computation*, 16(12): 2639–2664, 2004.

F. Pedregosa, G. Varoquaux, A. Gramfort, V. Michel, B. Thirion, O. Grisel, M. Blondel, P. Prettenhofer, R. Weiss, V. Dubourg, J. Vanderplas, A. Passos, D. Cournapeau, M. Brucher, M. Perrot, and E. Duchesnay. Scikit-learn: Machine learning in Python. *Journal of Machine Learning Research*, 12:2825–2830, 2011.

Jose Pinheiro, Douglas Bates, Saikat DebRoy, Deepayan Sarkar, and R Core Team. *nlme: Linear and Nonlinear Mixed Effects Models*, 2020. URL `https://CRAN.R-project.org/package=nlme`. R package version 3.1-147.

R Core Team. *R: A Language and Environment for Statistical Computing*. R Foundation for Statistical Computing, Vienna, Austria, 2020. URL `https://www.R-project.org/`.

Journal of Experimental Biology • Supplementary information
